# Mosquito Behaviour and Disease Control

**DOI:** 10.1093/emph/eou030

**Published:** 2014-11-27

**Authors:** Amber Gigi Hoi, Bernard D. Roitberg

**Affiliations:** Evolutionary and Behavioural Ecology Research Group, Department of Biological Sciences, Simon Fraser University, Burnaby, BC, Canada

## Mosquito control and disease management

Mosquitoes vector many important disease pathogens. Controls of mosquito-borne diseases often involve lowering the contact rate between human and vector, and killing the vector. For instance, long lasting insecticidal nets (LLINs) act on both of these levels. Unfortunately, these are at best short-term solutions as mosquitoes are known to exhibit resistance to these interventions. Physiological resistance to insecticides and repellents is well-documented worldwide [1]. However, behavioural resistance (e.g. changing encounter rates) to chemical threats and barriers is often underappreciated even though the importance of mosquito behaviour for disease control was emphasized as early as the 1950s [2]. For instance, it has been shown repeatedly that an increasing proportion of mosquitoes have started blood feeding earlier, before humans retire under their LLINs, essentially avoiding insecticides applied to the net’s surface [2].


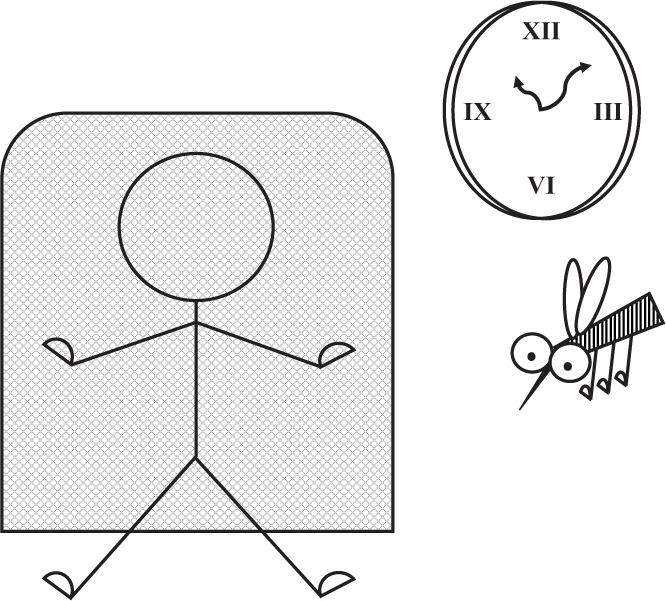


## Evolutionary perspectives

Observed changes in LLIN-associated blood-feeding behaviours in mosquitoes may arise from intervention-based selection, may already be present in their behavioural repertoire but are now expressed in the presence of LLINs, or may also be an artefact due to inappropriate measurements [2]. It is often difficult to distinguish between these causes in natural vector populations [2]. The bottom line is: mosquitoes are capable of highly flexible behaviour that can undermine intervention, but this flexibility is often constrained by trade-offs. For example, sugar and blood represent a dietary trade-off between somatic and gametic function for female mosquitoes. The former is primarily an energy source, and the latter is necessary for reproduction. These are non-substitutable, obligatory, resources, and mosquitoes can only feed to repletion from one at a time—the payoffs for doing so depend upon their energy state and size [3]. Spatially separating sugar sources, blood hosts and oviposition sites could, therefore, be effective in lowering malaria transmission intensity by forcing mosquitoes to reduce contact with humans [4]. These kinds of trade-offs are vulnerabilities in insect life history that humans could usefully exploit as it is very difficult to evolve out of them (unlike decreasing contact via LLINs, both sugar and blood resources are essential and mosquitoes would need to evolve a new digestive system to name the least!).

## Future implications

In designing mosquito management programmes, we should work with traits that are constrained by trade-offs such that resistance to intervention is not easily evolvable. These programmes should be designed on a case-by-case basis taking into account local micro-ecology. Much could be learned from the management of agricultural pests in this regard [5], such as push-pull strategies (use of sensory stimuli to lure pests away from crops and towards insect traps) that are employed worldwide for management of a variety of pests [6].
